# Classical Disease-Specific Autoantibodies in Systemic Sclerosis: Clinical Features, Gene Susceptibility, and Disease Stratification

**DOI:** 10.3389/fmed.2020.587773

**Published:** 2020-11-19

**Authors:** Changyi Yang, Shunli Tang, Dingxian Zhu, Yingguo Ding, Jianjun Qiao

**Affiliations:** Department of Dermatology, The First Affiliated Hospital, Zhejiang University School of Medicine, Hangzhou, China

**Keywords:** anti-topoisomerase antibodies, anticentromere antibodies, anti-RNA polymerase antibodies, systemic sclerosis, clinical manifestations, gene, disease stratification

## Abstract

Systemic sclerosis (SSc) is an autoimmune disease characterized by abnormalities in microcirculation, extracellular matrix accumulation, and immune activation. Autoantibodies are markers of immune abnormalities and provide diagnostic and predictive value in SSc. Anti-topoisomerase antibodies (ATAs), anticentromere antibodies (ACAs), and anti-RNA polymerase antibodies (ARAs) are the three classical specific antibodies with the highest availability and stability. In this review, we provide an overview of the recent progress in SSc research with respect to ATAs, ACAs, and ARAs, focusing on their application in distinguishing clinical phenotypes, such as malignancy and organ involvement, identifying genetic background in human leukocyte antigen (HLA) or non-HLA alleles, and their potential roles in disease pathogenesis based on the effects of antigen–antibody binding. We finally summarized the novel analysis using ATAs, ACAs, and ARAs on more detailed disease clusters. Considering these advantages, this review emphasizes that classical SSc-specific autoantibodies are still practical and have the potential for patient and risk stratification with applications in precise medicine for SSc.

## Introduction

Systemic sclerosis (SSc) or scleroderma is a chronic multi-system disease with heterogeneous manifestations (1). There is still a lack of recommendations with strong evidence regarding the diagnosis and management of several SSc-specific complications (2), leading to a reduced quality of life and an enormous burden for patients. The mechanism underlying SSc is characterized by three manifestations: vascular injury, immune abnormality, and fibrosis. Vascular injury is identified as an initial factor, whereas fibrosis is considered a sign of the end stage. Furthermore, immune activation has been proposed as a bridge throughout the disease course. Autoantibodies, indicators of immune abnormality, are detected in >90% of patients with SSc (3). Anti-topoisomerase antibodies (ATAs), anticentromere antibodies (ACAs), and anti-RNA polymerase antibodies (ARAs), first described in the 1970–1990s (4, 5), are the classical disease-specific autoantibodies (1).

Because of the high validity and reliability of ATAs, ACAs, and ARAs for SSc (6), the 2013 American College of Rheumatology/European League against Rheumatism (ACR/EULAR) SSc classification criteria included disease-specific autoantibodies as a scoring item (1), and the 2018 Japanese Dermatological Association listed them as minor diagnostic criteria (7). SSc-specific antibodies were also listed in the very early diagnosis of SSc (8) or UCTD-risk-SSc criteria (9). In general, the presence of these three SSc-specific autoantibodies may be relevant to the different clinical manifestations of SSc, such as diffuse/limited cutaneous subtypes and pulmonary fibrosis. Recently, bioinformatics helped discover new roles of these autoantibodies; genetic susceptibility analysis revealed the intrinsic characteristics of patients in different autoantibody subgroups (10). Moreover, cytology studies suggested pathological roles for ACAs, ATAs, and ARAs beyond disease diagnosis (11). Thus, the detection of ACAs, ATAs, and ARAs may facilitate the development of precise medicine.

For a systemic understanding of classical SSc-specific autoantibodies, we have reviewed the general information on ATAs, ACAs, and ARAs in clinical manifestations, emphasizing their role in SSc-related cancer. Next, we have comprehensively summarized research breakthroughs describing the genetic features of these autoantibodies, illustrated the potential pathogenesis pathway, and identified the novel disease clusters related to these SSc-specific autoantibodies.

## Classical Disease-Specific Autoantibodies in Clinical Manifestations

### Epidemiology

Although several studies have reported a varying prevalence of classical disease-specific autoantibodies in SSc, their reported sensitivity and specificity remain relatively stable (12). The prevalence of ATAs in patients with SSc was reported to be 14–71%, with a sensitivity of 24% and a specificity of 99.6% (1). ARAs were detected in 4–20% of patients, with 16% sensitivity and 97.5% specificity (13). The prevalence of ACAs in patients with SSc was 20–57.8%, with a sensitivity and specificity of 33 and 93%, respectively (13, 14). However, unlike ATAs and ARAs that are rarely detected in other autoimmune diseases, ACAs may be produced in systemic lupus erythematosus, Sjögren's syndrome, rheumatoid arthritis, and primary biliary cholangitis (15). Thus, the presence of ACAs in other disorders may help elucidate the occurrence trend of SSc overlap syndromes (16).

The levels of classical disease-specific autoantibodies reportedly vary in patients based on ethnicity. ACAs had a higher detection ratio in Hispanic and Caucasian patients compared with those belonging to African-American (*P* < 0.0001) and Asian ethnicities (*P* < 0.001) (14, 17). ATAs were mostly detected in Asian patients (17–19), whereas the prevalence levels of ARA were much higher in European (>10%) patients but lower in Asian (<6%) patients (14, 20).

### Clinical Associations

#### Skin Involvement

Among the classical autoantibodies, ACAs are more specific for the limited cutaneous subset of SSc (lcSSc) or CREST syndrome than ATAs (*P* = 0.005, OR = 2.54, 95% CI = 0.05–0.44) (21) and ARAs (*P* = 0.0005, OR = 0.13, 95% CI = 0.04–0.41); a longer disease duration before diagnosis (22) is related to good prognosis in terms of survival (23). Increased levels of ATAs are mainly associated with diffuse cutaneous disease (dcSSc) (*P* < 0.0001, OR = 4.26) (22) and serious organ involvement (13, 24). Patients with ATAs had higher SSc-related mortality rate and poor prognosis (25). ARA presence indicates a high risk of rapidly progressive skin thickening (*P* = 0.042, OR = 3.24, 95% CI = 1.44–7.31), and changes in ARA levels may correspond to changes in modified Rodnan skin thickness score (26, 27). A recent study revealed ARAs to be more prevalent in patients with sine scleroderma (P = 0.03) (28), an SSc subtype without cutaneous manifestations but with visceral involvement and serologic abnormalities that is difficult to diagnose (29). Since skin involvement was confirmed related to disease severity, different autoantibody groups can provide a preliminary grouping of patients for disease management.

#### Organ Involvement

ACAs are used to determine disease specificity in consistent vessel dysfunction not only for long-standing Raynaud's Phenomenon (RP) (*P* < 0.001) but also in pulmonary hypertension (PAH) without fibrosis (*P* < 0.001), compared with ATAs. Other vessel abnormalities include digital ulcers (*P* < 0.0001, OR = 0.50, 95% CI = 0.36–0.71), and a possible early/active nailfold videocapillaroscopy pattern (30). Furthermore, prior to a definite diagnosis of pulmonary diseases, ACAs were associated with a relatively rapid rise in pulmonary arterial systolic pressure and pulmonary vascular resistance (*P* < 0.001) (31). Thus, ACAs play a crucial role in consistent vascular injury. The appearance of ACAs at an early stage of SSc, related to vascular disease, should be closely monitored in patients, especially in the cardiopulmonary system.

Studies have shown ATA association with a higher probability of interstitial lung disease (ILD) (*P* < 0.0001, OR = 4.76, 95% CI = 3.48–6.50), even in ATA-positive patients with lcSSc (22, 25, 32). Recent studies have indicated that ATAs may be related to disability in hand, oral manifestation (33, 34), and flexion contractures in metacarpophalangeal and proximal interphalangeal joints (35), indicating their specificity, to a certain degree, in organ fibrosis. Therefore, early screening for organ involvement is recommended in ATA-positive patients because organ fibrosis is indicative of an irreversible stage.

A higher prevalence of musculoskeletal involvement, gastric antral vascular ectasia, ILD, PAH, and scleroderma renal crisis (SRC) has been reported in ARA-positive patients (26, 28, 36, 37). Notably, SRC was significantly more common in ARA-positive patients compared to ARA-negative ones (*P* < 0.0001). Moreover, ARAs showed high sensitivity (70.8%, 95% CI = 48.9–87.4), high specificity (87.8%, 95% CI = 84.3–90.8), and high negative predictive value (98.2%, 95% CI = 96.3–99.3) for patients with SRC. Interestingly, 16% of ARA-positive patients had a common history of silicone breast implants in a Japanese cohort (38, 39), suggesting a potential role of silicone in the development of disease with ARAs. In general, ARA measurement in patients with SSc is useful for diagnosis and risk stratification of severe manifestations, such as renal crisis and malignancy.

#### Malignancy

Similar to other autoimmune diseases, SSc is associated with malignancy in the lungs, breasts, liver, and hematologic systems. Although the role of autoantibodies is still under debate, ATAs, ACAs, or ARAs were barely detected in tumor-carrying patients without SSc (40).

ATAs were found to show higher risk of cancer after SSc diagnosis (HR = 1.4, 95% CI = 1.05–1.90, *P* = 0.0224) and have a significant negative impact on survival of the overall malignancy group (HR = 1.39, 95% CI = 1.08–01.80, *P* = 0.0106) (41). In a patient cohort with limited scleroderma/SSc overlap syndrome and mild organ involvement, ACAs correlated with a high risk of non-Hodgkin's lymphoma (42).

In contrast, ARAs are strongly associated with malignancy. Ami et al. first identified a strong association between RNAP I/III autoantibodies and malignancy contemporaneous with SSc (*P* = 0.027) (43). An Italian cohort study divided malignancy cases based on SSc onset: preceding (diagnosed >6 months before SSc onset), synchronous (6 months before to 12 months after), or metachronous (>12 months after); a significant association was observed between malignancies synchronous to SSc and ARA-positivity (OR = 7.38, 95% CI = 1.61–33.8) (44). Another large cohort study in the UK found breast cancer (>40%) to be the major malignancy subtype associated with SSc, and the frequency of cancer in ARA-positive patients was approximately twice that in the ATA- and ACA-positive groups (45). Similar findings (46–48) were reported in the Japanese and EUSTAR registries, further suggesting that ARA-positive patients with SSc shared similar pathological processes across different ethnicities. More recently, ARAs were shown to be an independent marker of coincident cancer and SSc irrespective of age (49). These results recommend a regular screening protocol for cancer in ARA-positive patients with SSc.

The relationship between these autoantibodies and malignancy provides new insights into cancer-risk stratification by clinical and serological phenotypes, thereby allowing targeted screening in this population.

### Classical Disease–Specific Autoantibodies and Genetic Characteristics

A specific genetic background with a combination of environmental and stochastic factors apparently contributes to SSc development (5, 50, 51). Autoantibodies are an essential part of the immune response; their susceptibility genes are not restricted to the major histocompatibility complex (human leukocyte antigen, HLA), but also include antigen presentation, lymphocyte activation, and cytokines/chemokines secretion (Tables 1, 2). Therefore, identifying the genetic background may provide a better understanding of SSc diagnosis, intrinsic classification, and therapeutic monitoring (73, 74).

**Table 1:** Publications of susceptible genes involved in lymphocyte activation in systemic sclerosis.

**Gene**	**Author, Year [References]**	**Research type**	**Case/Control**	**Locus/SNPs**	**Associated autoantibodies**	**Population**
STAT4	Krylov et al., 2017 (52)	Case–control	102/103	rs7574865 G/T	ATA	Russian
	Yi et al., 2013 (53)	Case–control	453/534	rs7574865 rs10168266	ATA	Han Chinese
	Dieudé et al., 2009 (54)	Case–control	440/485 (replication:445/485)	rs7574865 T	ATA	French Caucasian
PTPN22	Wipff et al., 2006 (55)	Case–control	121/103	PTPN22*R620W	No association	French Caucasian
	Balada et al., 2006 (56)	Case–control	54/55	PTPN22*R620W	No association	N/A
	Ramirez et al., 2012 (57)	Case–control	RA: 413 SLE: 94 SSc: 101 HC: 434	C1858T (rs2476601)	No association	Colombian
	Gourh et al., 2006 (58)	Case–control	White:850/430 Black:130/164 Hispanic:120/146 Choctaw Indian: 20/76	C1858T	ATA&ACA	US white, black, Hispanic, and Choctaw Indian individuals.
	Dieudé et al., 2008 (59)	Case–control & Meta–analysis	659/504	PTPN22 1858T	ATA	French Caucasian
	Diaz-Gallo et al., 2011 (60)	Meta–analysis	3422/3628	C1858T	ACA	Spain and 7 additional independent replication Caucasian
	Lee et al., 2012 (61)	Meta–analysis	4367/4771	C1858T	ACA	Multiple ethnicity
BANK1	Rueda et al., 2009 (62)	Case–control	2380/3270	rs10516487 G rs17266594 T rs3733197 G	ATA	Caucasian (American, Spanish, Dutch, German, Swedish and Italian)
	Dawidowicz et al., 2011 (63)	Case–control	900/1034	BANK1(N/A)	No association	European Caucasian

*NA, not available*.

**Table 2:** Publications of susceptible genes involved in inflammatory factors in systemic sclerosis.

**Gene**	**Author, Year [References]**	**Research**	**Case/Control**	**Locus/SNPs**	**Associated autoantibodies**	**Population**
TNF	Sato et al., 2004 (64)	Case-control	214/354	TNF-863A	ACA	UK white
	Lomelí-Nieto et al., 2019 (65)	Case-control	53/115	TNFA-308G>A TNFA-238G>A	ARA	Southern Mexico
AIF1	Alkassab et al., 2007 (66)	Case-control	1015/893	rs2269475 (T and CT/TT)	ACA	Caucasian African American Hispanic
IRF7	Carmona et al., 2012 (67)	Case-control	2316/2347	rs1131665 rs4963128 rs702966 rs2246614	ACA	USA Caucasian USA Spain
Th17	Rueda et al., 2009 (68)	Case-control	143/246 (replication:365/515)	IL23R	No association	Dutch Replication: Spanish
	Agarwal et al., 2009 (69)	Case-control	1402/1038	IL23R: rs11209026 rs11465804	ATA	N/A
	Mellal et al., 2018 (70)	Case-control	136/317	IL-21: rs6822844	ARA	Algerian
TNFSF	Coustet et al., 2012 (71)	Case-control	1031/1014	TNFSF4: rs2205960	ACA	French white
		Genotype-phenotype association analysis and Meta-analysis	4989/4661	TNFSF4: rs2205960	ACA	European white
	González et al., 2018 (72)	Case-control	4584/5160	TNFSF13B: rs374039502	No association	European

*NA, not available*.

### HLA and Classical Disease-Specific Autoantibodies

HLA alleles encode specific antigen-binding sequences, and thus play an essential role in antigen presentation, lymphocyte activation, and autoantibody production. HLA-class II (DRB1, DQB1, DQA1, and DPB1) alleles associated with SSc-related antibodies vary among different ethnic groups (Table 3).

**Table 3:** Antigen, prevalence, clinical features, and susceptible genotype of classical specific antibodies in systemic sclerosis.

**Autoantibody**	**Antigen**				**Prevalence (%)**			**Clinical features**		**Susceptible genotypes**	
	**Designation**	**Major**	**Location**	**Function**	**General**	**Early SSc (1)**	**VEDOSS (16)**	**Cutaneous subset**	**Special features**	**HLA alleles**	**Genes involved in pathways**
Anticentromere (ACAs)	Centromere proteins (CENP)	CENP -A, -B, -C	Around kinetochore	Constituent of the primary constriction of metaphase chromosomes	20–57.8 (13, 14, 75)	42.5–67.5	53.6	lcSSc (CREST syndrome)	Long-standing Raynaud's phenomenon PAH	DQB1^*^05:01/^*^26 DPB1^*^13:01 DRB1^*^07:01	TNF-863A AIF1 IRF7 TNFSF4 PTPN22
Anti-topoisomerase (ATAs)	DNA topoisomerase (Topo)	Topo I	Chromatin	Relaxation of supercoiled DNA	14–71 (20, 26, 76)	12.3–22.5	19.1–22	dcSSc	Cardiomyopathy IPF	DRB1^*^11:01/^*^11:04 DPB1^*^13:01, DRB1^*^15:02-DRB5^*^01:02 DPB1^*^09:01 DQB1^*^06:01 DRB1^*^08:04/DQA1^*^05:01	IL23R STAT4 PTPN22 BANK1 RXRB
Anti-RNA polymerase (ARAs)	RNA polymerase (RNAP)	RNAP I/III	Nucleoli nucleoplasm	Synthesis of ribosomal RNA precursors Synthesis of small RNAs	4–20 (13, 77)	0–31.3	N/A	dcSSc	Rapidly progressive skin thickening Musculoskeletal involvement, Gastric antral vascular ectasia, Tendon friction rubs, Synovitis, Myositis, Malignancy	DQB1^*^02:01 DRB1^*^04:05 DRB4^*^01 DQB1^*^04:01 DRB1^*^04:04 DRB1^*^11 DQB1^*^03 DRB1^*^08	TNFA-308G>A TNFA-238G>A IL-21

*NA, not available*.

ATAs were associated with DRB1^*^11:01/^*^11:04 in North-American Caucasians (*P* < 0.0001, OR = 6.93, 95% CI = 3.9–12.2); DPB1^*^13:01 in both African American (*P* < 0.001, OR = 4.3); and European-American patients (*P* = 1.47 × 10^−24^, OR = 13.7) (78); DRB1^*^15:02-DRB5^*^01:02, DPB1^*^09:01 haplotypes in Japanese and DQB1^*^06:01 in Chinese patients (78–81). Although DRB1^*^08:04, DQA1^*^05:01, and DPB1^*^13:01 were associated with African subjects, DPB1^*^13:01 showed the highest odds ratio.

ACAs were found associated with DQB1^*^05:01/^*^26 alleles (82). In Chinese Han patients, the expression of DQB1^*^05:01 was significantly increased (*P* = 1.6 × 10^−5^, OR = 3.4, 95% CI = 1.8–6.4), whereas in the European-American population, DPB1^*^13:01 and DRB1^*^07:01 alleles were more strongly relevant (*P* = 4.79 × 10^−20^, OR = 0.1) (78–80). The available data on African subjects are lacking, perhaps because of the small number of samples studied. DQB1^*^02:01 was first shown to be associated with RNAP I-III by Kuwana et al. (76). Another study proved the association between anti-RNAP I/III antibodies and DRB1^*^04:05 (*P* = 0.01, OR = 6.0, 95% CI = 1.4–25.2), DRB4^*^01 (*P* = 0.02, OR = 10.1, 95% CI = 1.4–74.1), and DQB1^*^04:01 (*P* = 0.01, OR = 6.0, 95% CI = 1.4–25.2) in Japanese patients (81). Recent evidence found that DRB1^*^04:04 (OR = 5.13), DRB1^*^11 (OR = 1.55), and DQB1^*^03 (OR = 2.38) alleles were more present in Hispanic and Caucasian patients, whereas DRB1^*^08 allele (OR = 3.92) was more present in African patients with ARAs (78, 79).

These findings indicate that specific HLA-alleles may provide susceptibility to classical disease-specific autoantibodies in SSc. Although the HLA associations in SSc patients with classical disease-specific autoantibodies remains unclear, these findings provide insights for the individual recognition of antibody specificities.

### Non-HLA Genes and Classical Disease-specific Autoantibodies

#### STAT4

Signal transducer and activator of transcription 4 (*STAT4*), a susceptibility gene for multiple autoimmune diseases, is associated with immune dysregulation, for example, in the imbalance of Th1/Th2 cytokine and the synthesis of the extracellular matrix across different ethnic groups (54, 83).

Dieudé et al. first identified *STAT4* polymorphism rs7574865 in association with ANAs (*P* = 0.01, OR = 1.30, 95% CI = 1.11–1.53) in SSc, although the specificity for ACAs/ATAs/ARAs was not confirmed (54). Another study in a Russian population indicated a possible association between ATAs and *rs7574865* (52). A large-cohort study demonstrated that *rs7574865* (*P* = 0.0012, OR = 0.56, 95% CI = 0.38–0.81) and *rs10168266* (*P* = 3.1 × 10^−4^, OR = 0.51, 95% CI = 0.35–0.75) were strongly associated with ATA presence and pulmonary fibrosis in Chinese patients with SSc (53).

*STAT4* is essential for the biological functions of various immune cells; however, its specific characteristics in SSc are unknown. Animal experiments have revealed that *STAT4*^−/−^ mice were resistant to SSc (84). Thus, these autoantibodies may provide a basis for a better understanding of the disease.

#### PTPN22

Protein tyrosine phosphatase N22 (*PTP22*) encodes a phosphatase related to the T-cell signaling pathway and shares a definite association with multiple autoimmune diseases. However, conflicting findings are reported in SSc.

Wipff et al. and Balada et al. demonstrated that PTPN22^*^620W was not associated with autoantibody patterns in a cohort of French Caucasian patients with SSc (55, 56). In contrast, Gourh et al. indicated that *PTPN22 R620W* polymorphism was associated with ACA- and ATA-positive subsets and was considered a risk factor in both Caucasian and African patients (58). It was suggested that a variation of *PTPN22* expression in the autoantibodies (ACAs or ATAs) was based on differences in ethnicities and presence of single-nucleotide polymorphism (SNP) (57, 59–61, 85).

#### BANK1

B-cell scaffold protein with ankyrin repeat gene (*BANK1*) encodes the substrate of LYN tyrosine kinase and participates in phosphorylation of triphosphate receptors, that are specifically expressed in B lymphocytes (63, 86, 87). Recent evidence suggests that *BANK1, IRF5*, and *STAT4* risk alleles display a multiplicatively increased risk of dcSSc (58, 62, 88, 89).

The first study to significantly implicate *BANK1* in SSc was reported in 2009; in 2,380 Caucasian patients with SSc, *BANK1* polymorphisms—*rs10516487, rs17266594*, and *rs3733197*—were found to be restricted to ATA-carrying subgroups (*P* = 0.03, OR = 1.20, 95% CI = 1.02–1.41; *P* = 0.01, OR = 1.24, 95% CI = 1.05–1.46; *P* = 0.004, OR = 1.26, 95% CI = 1.07–1.47, respectively) (90).

Notably, *BANK1* is chiefly expressed in CD19^+^ B cell-overexpressing patients with SSc (91). These findings may explain the role of abnormal B cells in SSc-specific autoantibody production.

#### TNF Alleles

Tumor necrosis factor (*TNF*), a key proinflammatory cytokine, plays an important role in SSc by upregulating Nuclear factor kappa B (92). Parks et al. first proposed that the *TNF-*β +*252* locus plays a crucial role in SSc etiopathogenesis (93). Other polymorphisms (*TNF-*α and *TNF receptor-II*) are also linked with autoantibodies in SSc (94). However, a linkage disequilibrium exists between *TNF* and HLA genes; therefore, the phenomenon may reflect the situation already described for HLA.

Several studies have attempted to elucidate this relationship. Extensive research has identified a strong primary association of *TNF-863A* and *TNF-1031C* alleles with ACA-positivity as well as *TNF-857T* allele with ATAs in SSc (64). Recent evidence indicated that *TNFA* polymorphisms, associated with higher sTNF-α levels, positively correlate with ARAs levels (65).

#### TNFSF

TNF (*TNFSF*) superfamily members *TNFSF13B*, encoding BAFF, and *TNFSF4*, encoding OX40 antigen ligand, are reportedly involved in SSc. Both play crucial roles in the interaction between T cells/antigen presentation and T- and B-cell activation (71, 72). Genotype–phenotype association analysis and meta-analysis confirmed *TNFSF4* as an SSc susceptibility gene and *rs2205960* as a putative causal variant with a preferential association with the ACA-positive SSc subtype (*P* = 0.0015, OR = 1.37, 95% CI = 1.12–1.66) (71).

*TNFSF4 rs1234214* is significantly associated with ACA-positivity (*P* = 0.005, OR = 1.33, 95% CI = 1.1–1.6) and ATA-positivity (*P* = 0.026, *OR* = 1.31, 95% CI = 1.02–1.7) (95). The association of *rs844648* with ARAs (*P* = 0.004, OR = 1.4, 95% CI = 1.1–1.8) was also confirmed (95).

Thus, *TNFSF4* may be involved in autoimmunity for the development of SSc.

#### AIF1

Allograft inflammatory factor 1 (*AIF1*) encodes a cytoplasmic calcium-binding protein that is present in damaged vessels of the lungs and skin lesions of patients with SSc, thereby presumably playing a role in vascular pathology (96–99).

Moreover, genetic association between *AIF1* polymorphism and the ACA-positive subset of SSc was confirmed (*P* = 0.006/0.002 in Caucasians/combined group, OR = 1.53/1.56 in Caucasians/combined group, 95% CI = 1.11–2.11/1.18–2.07 in Caucasians/combined group) (66). Limited by the absence of adequate data, confirmation of its potential biological relevance remains a significant challenge.

#### IRF7

Interferon regulatory factor 7 (*IRF7*), a member of the interferon regulatory transcription factor family and a key molecular determinant in interferon pathway, can activate type I interferon genes in response to viral agents or DNA/RNA-containing immune complex, first described by Carmona et al. (67).

IRF7 mRNA expression was significantly upregulated in the bleomycin-induced and tight-skin mouse models as well as in peripheral blood mononuclear cells and dermal fibroblasts from patients (100). Moreover, patients with different *IRF7* SNPs (rs1131665: *P* = 6.14 × 10^−4^, OR = 0.78; rs4963128: *P* = 6.14 × 10^−4^, OR = 0.79; rs702966: *P* = 3.83 × 10^−3^, OR = 0.82; and rs2246614: *P* = 3.83 × 10^−3^, OR = 0.83) were mostly related to ACA-positivity (67, 100, 101), thus supporting the fact that the *IRF7* locus represents a common risk factor for ACA production.

### Genes Associated With T-helper 17 Cell Pathway

Recent findings indicated the role of Th17 pathway in SSc, which is promoted by several factors including interleukin (IL)-17A, IL-17F, IL-21, and IL-23R (68, 70).

*IL23R* polymorphisms (rs11209026, rs11465804) were associated with susceptibility to ATA-positive SSc (*P* = 0.001, *P* = 0.0026, respectively) and considered protective against the development of PAH in patients with SSc (*P* = 3 × 10^−5^, *P* = 1 × 10^−5^, respectively). Additionally, an association between *IL-21 SNP (rs6822844)* and ARA production as well as digestive involvement (69) was found, indicating that Th17 genes were associated with SSc-susceptibility and specific-organ involvement (70).

### RXRB

A retinoid X receptor beta (*RXRB*) variant, *rs17847931*, is associated with antifibrotic activity in the skin and chromatin remodeling in ATA-positive patients with SSc (102). Since RXRB, a type of RXR, mediates the effects of retinoic acid that shows anti-fibrotic activity in skin tissues (103), the prospective therapeutic role of retinoic acid may be better applied in SSc groups with specific autoantibodies.

### Applications of Classical Disease-Specific Autoantibodies as Predictors of SSc Development

RP exists in more than 90% of patients with SSc and could precede organ fibrosis by years or even decades (104). However, RP without specificity is also found in the early stages of other autoimmune diseases. Importantly, patients with RP are at a risk of developing SSc.

SSc-specific autoantibodies independently predict definite SSc (105). Different autoantibodies were associated with a distinct time course of microvascular damage in a 20-year prospective study (105). ATAs were strongly predictive for SSc with a nine-fold probability of SSc occurrence in primary patients with RP (106). The presence of both ATAs and scleroderma patterns of nailfold capillaroscopy may increase the prediction accuracy and susceptibility (107–109).

Therefore, when patients present various clinical features and initial diagnosis is difficult, abnormal findings on these three SSc-specific autoantibodies could help distinguish SSc from early stages of other autoimmune diseases.

### As Biomarkers of Disease Phenotypes

ACAs, ATAs, and ARAs remain the most common SSc-specific autoantibodies in the majority of real-world studies. The use of these autoantibodies to define novel clinical classifications or disease clusters has been demonstrated over the years.

Moinzadeh et al. (107) used them to define five patient clusters with different clinical features: ATAs, strong ARAs, weak ARAs, ATAs, and others. Moreover, the statistical difference between the five clusters indicated that their use was not restricted to classification of the cutaneous subsets alone as previously reported. Further, Srivastava et al. (110) found that organ involvement was more associated with antibody profiles, whereas joint and vascular dysfunction were more related to cutaneous subsets.

Interestingly, the combination of ATAs and ACAs with cutaneous subsets or more parameters may predict outcomes better than their individual use. Nihtyanova et al. proposed seven groups of patients with SSc, combining autoantibody specificity and skin involvement (ATA + lcSSc, ATA + dcSSc, ACA + lcSSc, ARA+, other antibodies + lcSSc, other antibodies + dcSSc) (111) while Sobanski et al. (112) characterized six clusters based on antibody profiles (cutaneous subsets, organ damage, and prognosis together), thereby achieving a more precise risk stratification of patients. Similarly, an increased risk of cancer was found in ACA-positive patients with ACAs (113). Additionally, cancer-specific risk varied in different cutaneous subtypes, and the ARA + dcSSc group tended to have a greater risk of breast cancer, whereas the ARA + lcSSc group had a high risk of lung cancer.

In summary, ACAs, ATAs, and ARAs could be cost-effective screening tools for disease subclassification and would improve the management of patients with SSc, progressive SSc, and those at risk of developing it.

### As Initiators of Pathogenesis

Considering the limited treatment options and unpleasant outcomes for patients with SSc, a better understanding of its pathogenesis is required. As a bridge between vascular injury and irreversible fibrosis, autoantibodies may act as the actual pathogenetic agents, secondary consequences of tissue injury, or pure footprints of etiological operators.

**Figure 1:**
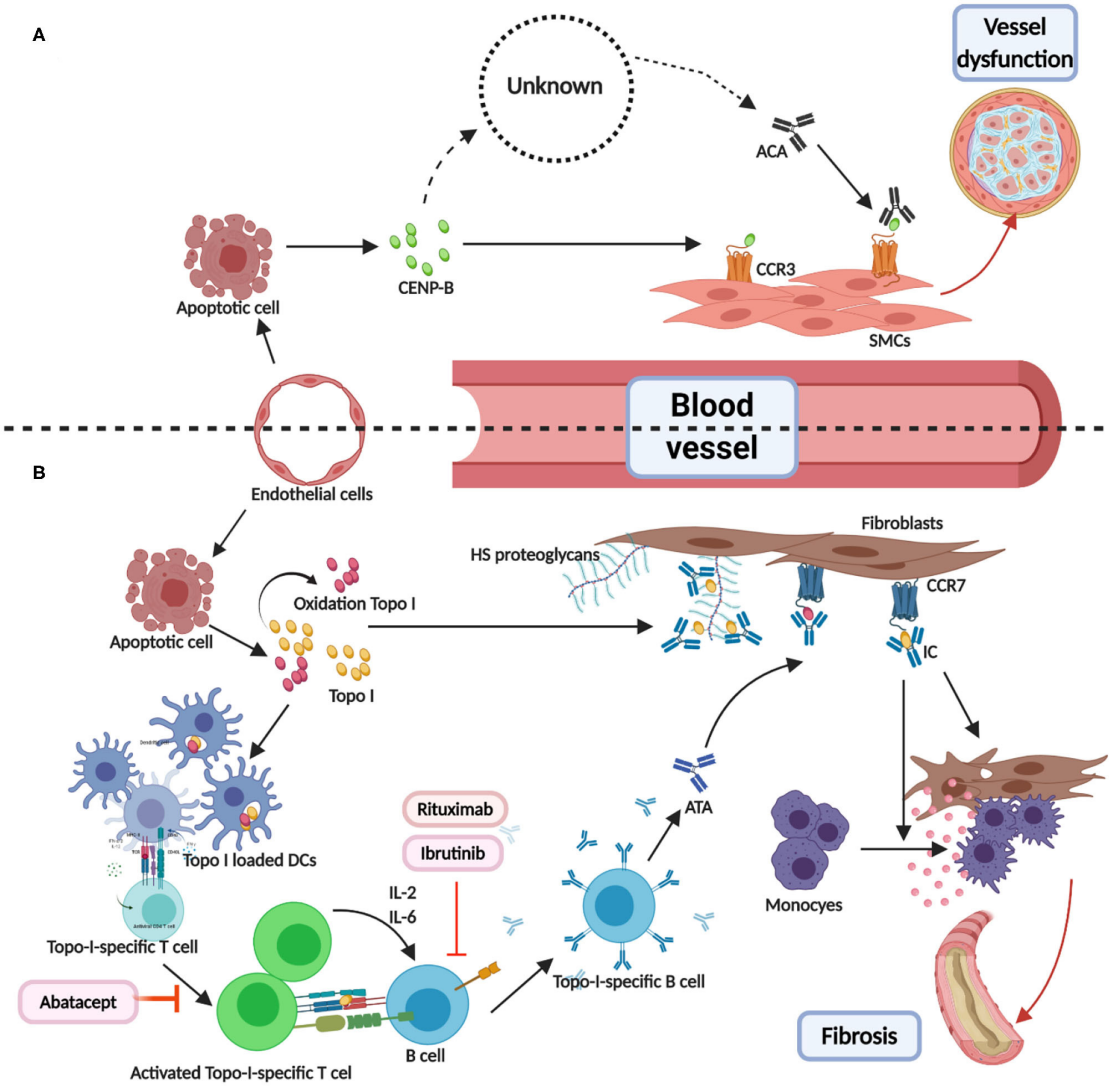
Direct combination of antibodies and antigens in systemic sclerosis. **(A)** CENP-B were released from the apoptotic ECs. Then, the extracellular CENP-B bound to the contractile-type PASMCs via CCR3. Next, the binding of CENP-B to the contractile SMCs stimulated migration in the wound healing assays. The exact way of production of ATAs was known. When combined with CCR3-binding CENP-B, ATAs may abolish vascular self-repair, further leading to angiopathy. **(B)** TOPO I was released from apoptotic ECs and some of them were oxidized to AOPP. Then, TOPO I was bound to the bystander fibroblasts via CCR7 or HS proteoglycans. DCs loaded with selected TOPO I could activate the intrinsic TOPO I–specific T cells. The activated special T cells produced IL-2 or IL-6 and communicated with B cells through the interactions of MHC-TCR and CD40-CD40L. T cell–dependent B cells were activated, thereby becoming TOPO I–specific B cells and resulting in ATAs. Binding TOPO I recruited circulating ATAs and composed ICs, which could induce the adhesion and activation of circulating monocytes. Abatacept-regulated dysfunction T cells. Rituximab and ibrutinib may be used as B-cell depletion therapy. CENP-B, centromere proteins B; EC, endothelial cell; PASMC, Pulmonary artery smooth muscle cells; CCR, CC chemokine receptor; SMC, smooth muscle cell; AOPP, advanced oxidation protein products; HS, heparan sulfate.

**Figure 2:**
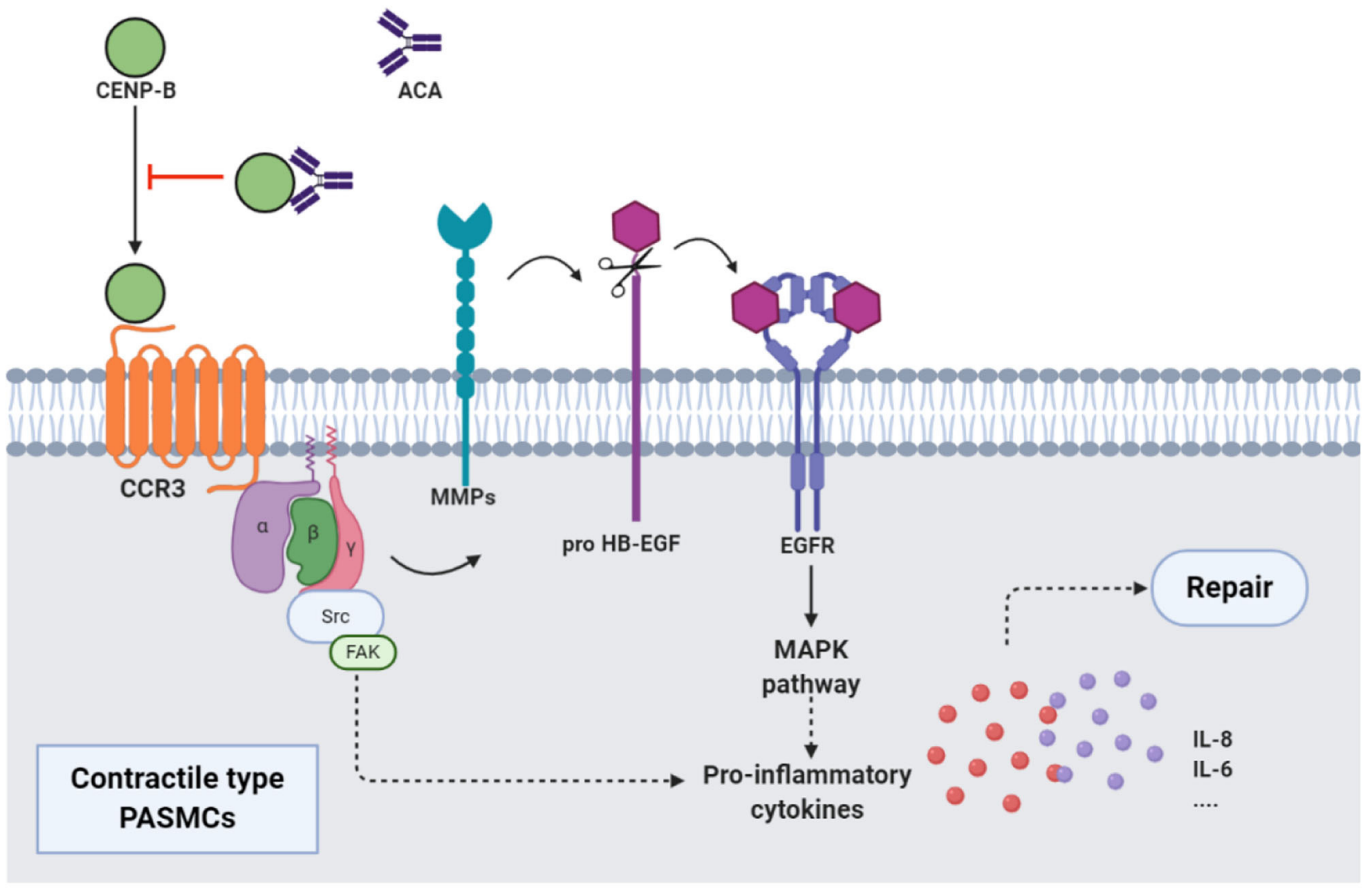
ACAs and CENP-B: CENP-B bound to CCR3. Then, the cross-talk between CCR3 and EGFR, which was mediated by the MMPs-dependent processing of pro HB-EGF, activated MAPK pathway, and production of proinflammatory cytokines such as IL-8, and promoted the migration of contractile-type PASMCs, further leading to vascular self-repair. ATAs from patients with SSc, when combined with CCR3-binding CENP-B, abolished the abovementioned pathway and inhibited the vascular self-repair. EGFR, epidermal growth factor receptor; MMP, matrix metalloproteinase; HB-EGF, heparin-binding EGF-like growth factor; MAPK, mitogen activated protein kinase.

**Figure 3:**
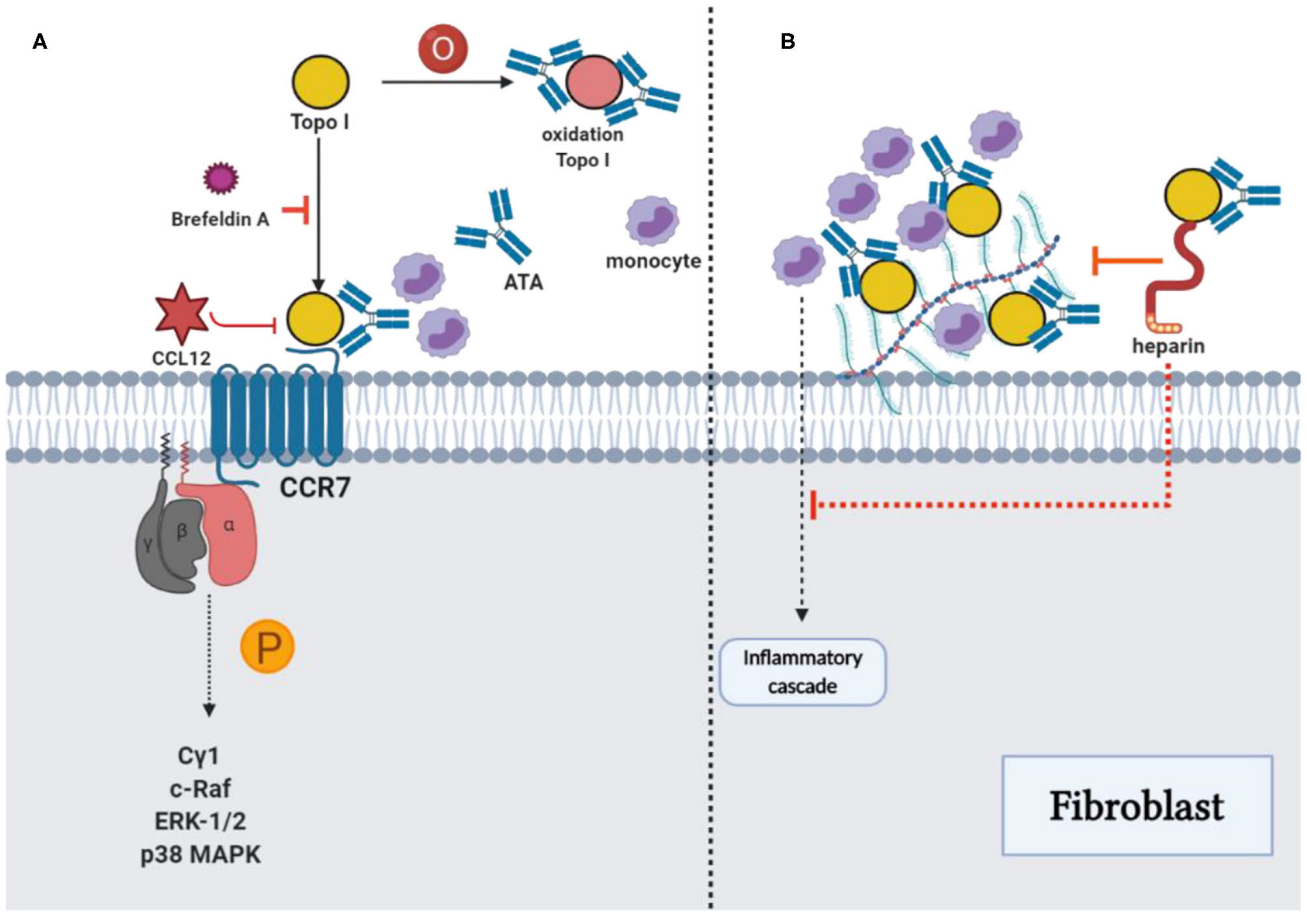
ATAs and topo I: Reinforcement of pathological functions. **(A)** The combination of TOPO I and fibroblasts could be suppressed by using brefeldin A, and oxidized TOPO I may have increased the antigenicity. The potential intracellular signaling pathway stimulated by TOPO I was the phosphorylation of phospholipase Cγ1, c-Raf, ERK-1/2, and p38 MAPK, which stimulated the migration of fibroblast. Cytokine-like effects of TOPO I in the pathway could be inhibited by CCL21. **(B)** TOPO I bound to HS proteoglycans on the fibroblast surface, as well as the accumulation of TOPO I on cell surfaces by ATAs could contribute to the initiation of an inflammatory cascade stimulating the fibrosis. The effect could be inhibited by heparin through the interference with TOPO I binding and the consequent accumulation of TOPO I-ATA ICs could be restrained with decreased monocyte adhesion, proinflammatory factors, and fibrosis.

ATAs and ACAs were found to participate in a pathological pathway involving endothelial cells injury and antigen release and presentation (114–117). The antigens (centromere proteins, topoisomerase, and RNA polymerase) for ACAs, ATAs, and ARAs are distributed in and around the nucleus, and play important roles in cellular structure and function. Therefore, the release of antigens, combination of antigens, and cell surface receptors, T- and B-cell collaboration (32), and antigen–antibody binding are interlinked and involved in disease occurrence, with a central role for the binding of antigens (topo I and CENP-B) (118, 119) and cell surface receptors (Chemokine Receptor 7 and Chemokine Receptor 3) (120–122), illustrated in Figure 1. We hypothesized two effects of the formation of immune complexes (ATA-topo I and ACA-CENP-B): reinforcement of pathological functions and inhibition of physiological functions. Figure 2 shows the pathway induced by the ACA-CENP-B complex and Figure 3 displays the pathway leading by ATA-topo I complex.

Three immune models with underlying distinct autoantibody signatures using multilayer profiling were identified (123). The ATA cluster showed a vascular phenotype with disrupted angiogenesis reflected by imbalanced antiangiogenic factors and cytokines such as IL-21 and sFLT-1. The ACA cluster showed a follicular T helper–B cell phenotype, characterized by low expression of inflammatory markers, such as IL-21, and relatively limited and mild clinical features. The ARA cluster showed a fibrotic phenotype, with Th2/Th17-mediated fibrosis by cytokines such as IL-17 and IL-21.

With advances in the detection of autoantibodies and underlying pathological markers, more precise targeting treatments, such as B-cell deletion, anti-cytokine antibodies, and vasodilators, may be developed for patients with different phenotypes.

## Conclusions and Remarks

In summary, although several other antibodies are reportedly associated with SSc, classical disease-specific autoantibodies are still considered significant for the diagnosis with extensive applicability.

With an increase in cross-sectional and longitudinal studies over the past few years, more specific clinical features in different antibody groups were identified, providing new insights into the risk-stratification of patients; this allowed targeted screening of patients with not only different cutaneous manifestations (diffuse/limited or sine scleroderma), but also a high risk of vital organ involvement, such as PAH, IPF, and SRC, and malignancy.

Since ATAs, ACAs, and ARAs show high validity and reliability among SSc autoantibodies, their application should not be limited to diagnosis and basic clinical classification. Moreover, clinical features, genes, and intrinsic characteristics can reflect the distinct autoantibody subtypes and ultimately reveal the underlying pathogenic pathways. Studies on genetic characteristics provide new insights for identifying disease-specific autoantibodies that may precede clinical symptoms and signs.

Taken together, the next step in the study of SSc classical disease-specific autoantibodies should include a wider range of stratification and precision medicine, such as risk prediction, disease cluster, and mechanism. Furthermore, research on the classical disease-specific autoantibodies in patients with SSc should be combined with genomes, proteomes, and metabolomes, and should be applied clinically.

## Author Contributions

CY analyzed and interpreted the data regarding autoantibodies of systemic sclerosis and the data from gene research works, and was a major contributor in writing the first manuscript. ST collected statistical data of studies in the revision (*p*-value, OR value, as well as 95% CI value) and proofread all references. DZ contributed to the language polish and corrected the grammatical errors, making a great contribution in writing the revised manuscript. YD contributed to the conception of the study and helped perform the analysis with constructive discussions. JQ contributed significantly to improve the review structure. All authors read and approved the final manuscript.

## Conflict of Interest

The authors declare that the research was conducted in the absence of any commercial or financial relationships that could be construed as a potential conflict of interest.

## Abbreviations

SSc: systemic sclerosis
ATAs: anti-topoisomerase antibodies
ACAs: anticentromere antibodies
ARAs: anti-RNA polymerase antibodies
ANA: antinuclear antibody
ECM: extracellular matrix
VEDOSS: very early diagnosis of SSc
UCTD: undifferentiated connective tissue disease
RP: Raynaud's phenomenon
CENP: centromere proteins
PAH: pulmonary hypertension
ILD: interstitial lung disease
SRC: scleroderma renal crisis
HLA: human leukocyte antigen
SNP: single-nucleotide polymorphism
STAT4: signal transducer and activator of transcription 4
PTP22: protein tyrosine phosphatase N22
BANK1: B-cell scaffold protein with ankyrin repeats gene
TNF: tumor necrosis factor
AIF1: allograft inflammatory factor 1
IRF: interferon regulatory transcription factor
PBMCs: peripheral blood mononuclear cells
IL: interleukin
TNFSF: tumor necrosis factor superfamily
EC: endothelial cells.

## References

[1] van den Hoogen F Khanna D Fransen J Johnson SR Baron M Tyndall A. 2013 classification criteria for systemic sclerosis: an American college of rheumatology/European league against rheumatism collaborative initiative. Arthritis Rheum. (2013) 65:2737–47. 10.1002/art.38098 24122180 PMC3930146

[2] Smith V Scirè CA Talarico R Airo P Alexander T Allanore Y. Systemic sclerosis: state of the art on clinical practice guidelines. RMD Open. (2018) 4(Suppl. 1):e000782. 10.1136/rmdopen-2018-000788 30402270 PMC6203100

[3] Stochmal A Czuwara J Trojanowska M Rudnicka L. Antinuclear antibodies in systemic sclerosis: an update. Clin Rev Allergy Immunol. (2020) 58:40–51. 10.1007/s12016-018-8718-8 30607749

[4] Moroi Y Peebles C Fritzler MJ Steigerwald J Tan EM. Autoantibody to centromere (kinetochore) in scleroderma sera. Proc Natl Acad Sci USA. (1980) 77:1627–31. 10.1073/pnas.77.3.1627 6966403 PMC348550

[5] Douvas AS Achten M Tan EM. Identification of a nuclear protein (Scl-70) as a unique target of human antinuclear antibodies in scleroderma. J Biol Chem. (1979) 254:10514–22. 10.1002/chem.200903108 385602

[6] Liaskos C Marou E Simopoulou T Barmakoudi M Efthymiou G Scheper T. Disease-related autoantibody profile in patients with systemic sclerosis. Autoimmunity. (2017) 50:414–21. 10.1080/08916934.2017.1357699 28749191

[7] Asano Y Jinnin M Kawaguchi Y Kuwana M Goto D Sato S. Diagnostic criteria, severity classification and guidelines of systemic sclerosis. J Dermatol. (2018) 45:633–91. 10.1111/1346-8138.14162 29687465

[8] Valentini G. Undifferentiated connective tissue disease at risk for systemic sclerosis (SSc) (so far referred to as very early/early SSc or pre-SSc). Autoimmun Rev. (2015) 14:210–13. 10.1016/j.autrev.2014.11.002 25461837

[9] Bellando-Randone S Matucci-Cerinic M. Very early systemic sclerosis and pre-systemic sclerosis: definition, recognition, clinical relevance and future directions. Curr Rheumatol Rep. (2017) 19:65. 10.1007/s11926-017-0684-2 28921059

[10] Sobanski V Giovannelli J Riemekasten G Airo P Vettori S Cozzi F. Phenotypes determined by cluster analysis and their survival in the prospective eustar cohort of patients with systemic sclerosis. Ann Rheum Dis. (2015) 74:90. 10.1136/annrheumdis-2015-eular.191530969034

[11] Sobanski V Lescoat A Launay D. Novel classifications for systemic sclerosis: challenging historical subsets to unlock new doors. Curr Opin Rheumatol. (2020) 32:463–71. 10.1097/BOR.0000000000000747 32941248

[12] Denton CP Khanna D. Systemic sclerosis. Lancet. (2017) 390:1685–99. 10.1016/S0140-6736(17)30933-9 28413064

[13] Meyer O. How useful are serum autoantibodies in the diagnosis and prognosis of systemic sclerosis? Clin Rheumatol. (1998) 17:179–80. 10.1007/BF01451042 9694047

[14] Nandiwada SL Peterson LK Mayes MD Jaskowski TD Malmberg E Assassi S. Ethnic differences in autoantibody diversity and hierarchy: more clues from a US cohort of patients with systemic sclerosis. J Rheumatol. (2016) 43:1816–24. 10.3899/jrheum.160106 27481902

[15] Liberal R Grant CR Sakkas L Bizzaro N Bogdanos DP. Diagnostic and clinical significance of anti-centromere antibodies in primary biliary cirrhosis. Clin Res Hepatol Gastroenterol. (2013) 37:572–85. 10.1016/j.clinre.2013.04.005 23876351

[16] Tsukamoto M Suzuki K Takeuchi T. Initial presentation determines clinical entity in patients with anti-centromere antibody positivity. Int J Rheum Dis. (2019) 22:103–7. 10.1111/1756-185X.13439 30428504

[17] Jaeger VK Tikly M Xu D Siegert E Hachulla E Airò P. Racial differences in systemic sclerosis disease presentation: a European scleroderma trials and research group study. Rheumatology. (2020) 59:1684–94. 10.1093/rheumatology/kez486 31680161

[18] Foocharoen C Watcharenwong P Netwijitpan S Mahakkanukrauh A Suwannaroj S Nanagara R. Relevance of clinical and autoantibody profiles in systemic sclerosis among thais. Int J Rheum Dis. (2017) 20:1572–81. 10.1111/1756-185X.13060 28296274

[19] Utsunomiya A Hasegawa M Oyama N Asano Y Endo H Fujimoto M Clinical course of Japanese patients with early systemic sclerosis: a multicenter, prospective, observational study. Modern Rheumatol. (2020) 21:1–9. 10.1080/14397595.2020.1751408 32243215

[20] Wang J Assassi S Guo G Tu W Wu W Yang L. Clinical and serological features of systemic sclerosis in a Chinese cohort. Clin Rheumatol. (2013) 32:617–21. 10.1007/s10067-012-2145-7 23271609 PMC3734856

[21] Foocharoen C Suwannachat P Netwijitpan S Mahakkanukrauh A Suwannaroj S Nanagara R. Clinical differences between Thai systemic sclerosis patients with positive versus negative anti-topoisomerase I. Int J Rheum Dis. (2016) 19:312–20. 10.1111/1756-185X.12492 25293362

[22] Mierau R Moinzadeh P Riemekasten G Melchers I Meurer M Reichenberger F. Frequency of disease-associated and other nuclear autoantibodies in patients of the German network for systemic scleroderma: correlation with characteristic clinical features. Arthritis Res Ther. (2011) 13:R172. 10.1186/ar3495 22018289 PMC3308107

[23] Miyawaki S Asanuma H Nishiyama S Yoshinaga Y. Clinical and serological heterogeneity in patients with anticentromere antibodies. J Rheumatol. (2005) 32:1488–94. 10.1097/01.rhu.0000173620.95740.e2 16078324

[24] Ferri C Giuggioli D Guiducci S Lumetti F Bajocchi G Magnani L Systemic sclerosis progression INvestiGation (SPRING) Italian registry: demographic and clinico-serological features of the scleroderma spectrum. Clin Exp Rheumatol. (2020) 125:40–47. Available online at: 24 https://www.clinexprheumatol.org/article.asp?a=14762 32301427

[25] Kranenburg P van den Hombergh WM Knaapen-Hans HK van den Hoogen FH Fransen J Vonk MC. Survival and organ involvement in patients with limited cutaneous systemic sclerosis and anti-topoisomerase-I antibodies: determined by skin subtype or auto-antibody subtype? A long-term follow-up study. Rheumatology. (2016) 55:2001–8. 10.1093/rheumatology/kew298 27520796

[26] Hamaguchi Y Kodera M Matsushita T Hasegawa M Inaba Y Usuda T. Clinical and immunologic predictors of scleroderma renal crisis in Japanese systemic sclerosis patients with anti-RNA polymerase III autoantibodies. Arthritis Rheumatol. (2015) 67:1045–52. 10.1002/art.38994 25512203

[27] Terras S Hartenstein H Höxtermann S Gambichler T Kreuter A. RNA polymerase III autoantibodies may indicate renal and more severe skin involvement in systemic sclerosis. Int J Dermatol. (2016) 55:882–5. 10.1111/ijd.13032 26499848

[28] Callejas-Moraga EL Guillén-Del-Castillo A Marín-Sánchez AM Roca-Herrera M Balada E Tolosa-Vilella C. Clinical features of systemic sclerosis patients with anti-RNA polymerase III antibody in a single centre in Spain. Clin Exp Rheumatol. (2019) 119:41–48. Available online at: https://www.clinexprheumatol.org/article.asp?a=13278 30767873

[29] Kucharz EJ Kopeć-Medrek M. Systemic sclerosis sine scleroderma. Adv Clin Exp Med. (2017) 26:875–80. 10.17219/acem/64334 29068586

[30] Sulli A Ruaro B Smith V Pizzorni C Zampogna G Gallo M. Progression of nailfold microvascular damage and antinuclear antibody pattern in systemic sclerosis. J Rheumatol. (2013) 40:634–9. 10.3899/jrheum.121089 23457379

[31] Gunn J Pauling JD McHugh NJ. Impact of anti-centromere antibodies on pulmonary function test results in patients with systemic sclerosis without established or suspected pulmonary disease. Clin Rheumatol. (2014) 33:869–71. 10.1007/s10067-014-2616-0 24752346

[32] Fava A Cimbro R Wigley FM Liu QR Rosen A Boin F. Frequency of circulating topoisomerase-I-specific CD4 T cells predicts presence and progression of interstitial lung disease in scleroderma. Arthritis Res Ther. (2016) 18:99. 10.1186/s13075-016-0993-2 27145754 PMC4857293

[33] Jaeger VK Wirz EG Allanore Y Rossbach P Riemekasten G Hachulla E. Incidences and risk factors of organ manifestations in the early course of systemic sclerosis: a longitudinal EUSTAR study. PLoS ONE. (2016) 11:e0163894. 10.1371/journal.pone.0163894 27706206 PMC5051961

[34] Bajraktari IH Kryeziu A Sherifi F Bajraktari H Lahu A Bajraktari G. Oral manifestations of systemic sclerosis and correlation with anti-topoisomerase I antibodies (SCL-70). Med Arch. (2015) 69:153–6. 10.5455/medarh.2015.69.153-156 26261381 PMC4500385

[35] Radić M Martinović Kaliterna D Ljutić D. The level of anti-topoisomerase I antibodies highly correlates with metacarpophalangeal and proximal interphalangeal joints flexion contractures in patients with systemic sclerosis. Clin Exp Rheumatol. (2006) 24:407–12. Available online at: https://www.clinexprheumatol.org/abstract.asp?a=2895 16956431

[36] Bhavsar SV Carmona R. Anti-RNA polymerase III antibodies in the diagnosis of scleroderma renal crisis in the absence of skin disease. J Clin Rheumatol. (2014) 20:379–82. 10.1097/RHU.0000000000000167 25275766

[37] Hoffmann-Vold AM Midtvedt Ø Tennøe AH Garen T Lund MB Aaløkken TM. Cardiopulmonary disease development in Anti-RNA polymerase III-positive systemic sclerosis: comparative analyses from an unselected, prospective patient cohort. J Rheumatol. (2017) 44:459–65. 10.3899/jrheum.160867 28089974

[38] Dall'Ara F Lazzaroni MG Antonioli CM Airò P. Systemic sclerosis with anti-RNA polymerase III positivity following silicone breast implant rupture: possible role of B-cell depletion and implant removal in the treatment. Rheumatol Int. (2017) 37:847–51. 10.1007/s00296-017-3654-0 28160072

[39] Saigusa R Asano Y Nakamura K Yamashita T Ichimura Y Takahashi T. Association of anti-RNA polymerase III antibody and silicone breast implants in patients with systemic sclerosis. J Dermatol. (2016) 43:808–10. 10.1111/1346-8138.13292 26876001

[40] Shah AA Rosen A Hummers LK May BJ Kaushiva A Roden RBS Evaluation of cancer-associated myositis and scleroderma autoantibodies in breast cancer patients without rheumatic disease. Clin Exp Rheumatol. (2017) 106:71–74. Available online at: https://www.clinexprheumatol.org/abstract.asp?a=11468 PMC5947318 28628466

[41] Watad A McGonagle D Bragazzi NL Tiosano S Comaneshter D Shoenfeld Y. Autoantibody status in systemic sclerosis patients defines both cancer risk and survival with ANA negativity in cases with concomitant cancer having a worse survival. Oncoimmunology. (2019) 8:e1588084. 10.1080/2162402X.2019.1588084 31069155 PMC6492983

[42] Baldini C Mosca M Della Rossa A Pepe P Notarstefano C Ferro F. Overlap of ACA-positive systemic sclerosis and Sjögren's syndrome: a distinct clinical entity with mild organ involvement but at high risk of lymphoma. Clin Exp Rheumatol. (2013) 31:272–80. Available online at: https://www.clinexprheumatol.org/abstract.asp?a=6520 23343785

[43] Shah AA Rosen A Hummers L Wigley F Casciola-Rosen L. Close temporal relationship between onset of cancer and scleroderma in patients with RNA polymerase I/III antibodies. Arthritis Rheum. (2010) 62:2787–95. 10.1002/art.27549 20506513 PMC2946521

[44] Airo P Ceribelli A Cavazzana I Taraborelli M Zingarelli S Franceschini F. Malignancies in Italian patients with systemic sclerosis positive for anti-RNA polymerase III antibodies. J Rheumatol. (2011) 38:1329–34. 10.3899/jrheum.101144 21459934

[45] Moinzadeh P Fonseca C Hellmich M Shah AA Chighizola C Denton CP. Association of anti-RNA polymerase III autoantibodies and cancer in scleroderma. Arthritis Res Ther. (2014) 16:R53. 10.1186/ar4486 24524733 PMC3978927

[46] Motegi S Toki S Yamada K Uchiyama A Ishikawa O. Demographic and clinical features of systemic sclerosis patients with anti-RNA polymerase III antibodies. J Dermatol. (2015) 42:189–92. 10.1111/1346-8138.12722 25483258

[47] Saigusa R Asano Y Nakamura K Miura S Ichimura Y Takahashi T. Association of anti-RNA polymerase III antibody and malignancy in Japanese patients with systemic sclerosis. J Dermatol. (2015) 42:524–7. 10.1111/1346-8138.12827 25720827

[48] Lazzaroni MG Cavazzana I Colombo E Dobrota R Hernandez J Hesselstrand R. Malignancies in patients with anti-RNA polymerase III antibodies and systemic sclerosis: analysis of the EULAR scleroderma trials and research cohort and possible recommendations for screening. J Rheumatol. (2017) 44:639–47. 10.3899/jrheum.160817 28089973

[49] Maria ATJ Partouche L Goulabchand R Rivière S Rozier P Bourgier C. Intriguing relationships between cancer and systemic sclerosis: role of the immune system and other contributors. Front Immunol. (2018) 9:3112. 10.3389/fimmu.2018.03112 30687318 PMC6335319

[50] Arnett FC. HLA and autoimmunity in scleroderma (systemic sclerosis). Int Rev Immunol. (1995) 12:107–28. 10.3109/08830189509056707 7650416

[51] Mayes MD. Scleroderma epidemiology. Rheum Dis Clin North Am. (1996) 22:751–64. 10.1016/S0889-857X(05)70299-4 8923594

[52] Krylov MY Ananyeva LP Koneva O Starovoytova MN Desinova OV Ovsyannikova OB. [The influence of STAT4 rs7574865 (G/T) polymorphism on the risk of clinical and immunological phenotypes of systemic sclerosis in a Russian patient population: results of a pilot study]. Terapevticheskii Arkhiv. (2017) 89:20–25. 10.17116/terarkh201789520-25 28631694

[53] Yi L Wang JC Guo XJ Gu YH Tu WZ Guo G. STAT4 is a genetic risk factor for systemic sclerosis in a Chinese population. Int J Immunopathol Pharmacol. (2013) 26:473–8. 10.1177/039463201302600220 23755762 PMC4105920

[54] Dieudé P Guedj M Wipff J Ruiz B Hachulla E Diot E. STAT4 is a genetic risk factor for systemic sclerosis having additive effects with IRF5 on disease susceptibility and related pulmonary fibrosis. Arthritis Rheum. (2009) 60:2472–9. 10.1002/art.24688 19644887

[55] Wipff J Allanore Y Kahan A Meyer O Mouthon L Guillevin L. Lack of association between the protein tyrosine phosphatase non-receptor 22 (PTPN22)^*^620W allele and systemic sclerosis in the French Caucasian population. Ann Rheum Dis. (2006) 65:1230–2. 10.1136/ard.2005.048181 16464986 PMC1798267

[56] Balada E Simeón-Aznar CP Serrano-Acedo S Martínez-Lostao L Selva-O'Callaghan A Fonollosa-Pla V. Lack of association of the PTPN22 gene polymorphism R620W with systemic sclerosis. Clin Exp Rheumatol. (2006) 24:321–4. Available online at: https://www.clinexprheumatol.org/abstract.asp?a=2877 16870103

[57] Ramirez M Quintana G Diaz-Gallo LM Caminos J Garces M Cepeda L. The PTPN22 C1858T variant as a risk factor for rheumatoid arthritis and systemic lupus erythematosus but not for systemic sclerosis in the Colombian population. Clin Exp Rheumatol. (2012) 30:520–4. Available online at: https://www.clinexprheumatol.org/abstract.asp?a=4638 22704547

[58] Gourh P Tan FK Assassi S Ahn CW McNearney TA Fischbach M. Association of the PTPN22 R620W polymorphism with anti-topoisomerase I- and anticentromere antibody-positive systemic sclerosis. Arthritis Rheum. (2006) 54:3945–53. 10.1002/art.22196 17133608

[59] Dieudé P Guedj M Wipff J Avouac J Hachulla E Diot E. The PTPN22 620W allele confers susceptibility to systemic sclerosis: findings of a large case-control study of European Caucasians and a meta-analysis. Arthritis Rheum. (2008) 58:2183–8. 10.1002/art.23601 18576360

[60] Diaz-Gallo LM Gourh P Broen J Simeon C Fonollosa V Ortego-Centeno N. Analysis of the influence of PTPN22 gene polymorphisms in systemic sclerosis. Ann Rheum Dis. (2011) 70:454–62. 10.1136/ard.2010.130138 21131644 PMC3170726

[61] Lee YH Choi SJ Ji JD Song GG. The association between the PTPN22 C1858T polymorphism and systemic sclerosis: a meta-analysis. Mol Biol Rep. (2012) 39:3103–8. 10.1007/s11033-011-1074-x 21688149

[62] Rueda B Broen J Simeon C Hesselstrand R Diaz B Suárez H. The STAT4 gene influences the genetic predisposition to systemic sclerosis phenotype. Hum Mol Genet. (2009) 18:2071–7. 10.1093/hmg/ddp119 19286670

[63] Dawidowicz K Dieudé P Avouac J Wipff J Hachulla E Diot E. Association study of B-cell marker gene polymorphisms in European Caucasian patients with systemic sclerosis. Clin Exp Rheumatol. (2011) 29:839–42. Available online at: https://www.clinexprheumatol.org/abstract.asp?a=4498 21961844

[64] Sato H Lagan AL Alexopoulou C Vassilakis DA Ahmad T Pantelidis P. The TNF-863A allele strongly associates with anticentromere antibody positivity in scleroderma. Arthritis Rheum. (2004) 50:558–64. 10.1002/art.20065 14872499

[65] Lomelí-Nieto JA Muñoz-Valle JF Baños-Hernández CJ Navarro-Zarza JE Ramírez-Dueñas MG Sánchez-Hernández PE. TNFA−308G>A and−238G>A polymorphisms and risk to systemic sclerosis: impact on TNF-α serum levels, TNFA mRNA expression, and autoantibodies. Clin Exp Med. (2019) 19:439–47. 10.1007/s10238-019-00569-4 31353423

[66] Alkassab F Gourh P Tan FK McNearney T Fischbach M Ahn C. An allograft inflammatory factor 1 (AIF1) single nucleotide polymorphism (SNP) is associated with anticentromere antibody positive systemic sclerosis. Rheumatology. (2007) 46:1248–51. 10.1093/rheumatology/kem057 17522098

[67] Carmona FD Gutala R Simeón CP Carreira P Ortego-Centeno N Vicente-Rabaneda E. Novel identification of the IRF7 region as an anticentromere autoantibody propensity locus in systemic sclerosis. Ann Rheum Dis. (2012) 71:114–9. 10.1136/annrheumdis-2011-200275 21926187 PMC3369428

[68] Rueda B Broen J Torres O Simeon C Ortego-Centeno N Schrijvenaars MM. The interleukin 23 receptor gene does not confer risk to systemic sclerosis and is not associated with systemic sclerosis disease phenotype. Ann Rheum Dis. (2009) 68:253–6. 10.1136/ard.2008.096719 18713787

[69] Agarwal SK Gourh P Shete S Paz G Divecha D Reveille JD. Association of interleukin 23 receptor polymorphisms with anti-topoisomerase-I positivity and pulmonary hypertension in systemic sclerosis. J Rheumatol. (2009) 36:2715–23. 10.3899/jrheum.090421 19918037 PMC2895677

[70] Mellal Y Allam I Tahiat A Abessemed A Nebbab R Ladjouze A. Th17 pathway genes polymorphisms in Algerian patients with systemic sclerosis. Acta Reumatol Portuguesa. (2018) 43:269–78. 30641535

[71] Coustet B Bouaziz M Dieudé P Guedj M Bossini-Castillo L Agarwal S. Independent replication and meta analysis of association studies establish TNFSF4 as a susceptibility gene preferentially associated with the subset of anticentromere-positive patients with systemic sclerosis. J Rheumatol. (2012) 39:997–1003. 10.3899/jrheum.111270 22422496 PMC3687343

[72] González-Serna D Carmona EG Ortego-Centeno N Simeón CP Solans R Hernández-Rodríguez J. A TNFSF13B functional variant is not involved in systemic sclerosis and giant cell arteritis susceptibility. PLoS ONE. (2018) 13:e0209343. 10.1371/journal.pone.0209343 30586461 PMC6306228

[73] Hinchcliff M Mahoney JM. Towards a new classification of systemic sclerosis. Nat Rev Rheumatol. (2019) 15:456–7. 10.1038/s41584-019-0257-z 31217541

[74] Chairta P Nicolaou P Christodoulou K. Genomic and genetic studies of systemic sclerosis: a systematic review. Hum Immunol. (2017) 78:153–65. 10.1016/j.humimm.2016.10.017 27984087

[75] Mahler M Maes L Blockmans D Westhovens R Bossuyt X Riemekasten G. Clinical and serological evaluation of a novel CENP-A peptide based ELISA. Arthritis Res Ther. (2010) 12:R99. 10.1186/ar3029 20487535 PMC2911886

[76] Kuwana M Okano Y Kaburaki J Medsger TA Jr Wright TM. Autoantibodies to RNA polymerases recognize multiple subunits and demonstrate cross-reactivity with RNA polymerase complexes. Arthritis Rheum. (1999) 42:275–84. 10.1002/1529-0131(199902)42:2<275::AID-ANR9>3.0.CO;2-P 10025921

[77] Nihtyanova SI Parker JC Black CM Bunn CC Denton CP. A longitudinal study of anti-RNA polymerase III antibody levels in systemic sclerosis. Rheumatology. (2009) 48:1218–21. 10.1093/rheumatology/kep215 19696067

[78] Gourh P Safran SA Alexander T Boyden SE Morgan ND Shah AA. HLA and autoantibodies define scleroderma subtypes and risk in African and European Americans and suggest a role for molecular mimicry. Proc Natl Acad Sci U S A. (2020) 117:552–62. 10.1073/pnas.1906593116 31871193 PMC6955366

[79] Arnett FC Gourh P Shete S Ahn CW Honey RE Agarwal SK. Major histocompatibility complex (MHC) class II alleles, haplotypes and epitopes which confer susceptibility or protection in systemic sclerosis: analyses in 1300 Caucasian, African-American and Hispanic cases and 1000 controls. Ann Rheum Dis. (2010) 69:822–7. 10.1136/ard.2009.111906 19596691 PMC2916702

[80] Zhou XD Yi L Guo XJ Chen E Zou HJ Jin L. Association of HLA-DQB1^*^0501 with scleroderma and its clinical features in Chinese population. Int J Immunopathol Pharmacol. (2013) 26:747–51. 10.1177/039463201302600318 24067471 PMC3887513

[81] Kuwana M Pandey JP Silver RM Kawakami Y Kaburaki J. HLA class II alleles in systemic sclerosis patients with anti-RNA polymerase I/III antibody: associations with subunit reactivities. J Rheumatol. (2003) 30:2392–7. Available online at: https://www.jrheum.org/content/30/11/2392.long 14677183

[82] McHugh NJ Whyte J Artlett C Briggs DC Stephens CO Olsen NJ. Anti-centromere antibodies (ACA) in systemic sclerosis patients and their relatives: a serological and HLA study. Clin Exp Immunol. (1994) 96:267–74. 10.1111/j.1365-2249.1994.tb06552.x 8187334 PMC1534886

[83] Xu Y Wang W Tian Y Liu J Yang R. Polymorphisms in STAT4 and IRF5 increase the risk of systemic sclerosis: a meta-analysis. Int J Dermatol. (2016) 55:408–16. 10.1111/ijd.12839 26712637

[84] Avouac J Fürnrohr BG Tomcik M Palumbo K Zerr P Horn A. Inactivation of the transcription factor STAT-4 prevents inflammation-driven fibrosis in animal models of systemic sclerosis. Arthritis Rheum. (2011) 63:800–9. 10.1002/art.30171 21360510

[85] Tizaoui K Kim SH Jeong GH Kronbichler A Lee KS Lee KH. Association of PTPN22 1858C/T polymorphism with autoimmune diseases: a systematic review and bayesian approach. J Clin Med. (2019) 8:347. 10.3390/jcm8030347 30871019 PMC6462981

[86] Kozyrev SV Abelson AK Wojcik J Zaghlool A Linga Reddy MV Sanchez E. Functional variants in the B-cell gene BANK1 are associated with systemic lupus erythematosus. Nat Genet. (2008) 40:211–16. 10.1038/ng.79 18204447

[87] Orozco G Abelson AK González-Gay MA Balsa A Pascual-Salcedo D García A. Study of functional variants of the BANK1 gene in rheumatoid arthritis. Arthritis Rheum. (2009) 60:372–9. 10.1002/art.24244 19180476

[88] Dieudé P Wipff J Guedj M Ruiz B Melchers I Hachulla E. BANK1 is a genetic risk factor for diffuse cutaneous systemic sclerosis and has additive effects with IRF5 and STAT4. Arthritis Rheum. (2009) 60:3447–54. 10.1002/art.24885 19877059

[89] Coustet B Dieudé P Guedj M Bouaziz M Avouac J Ruiz B. C8orf13-BLK is a genetic risk locus for systemic sclerosis and has additive effects with BANK1: results from a large French cohort and meta-analysis. Arthritis Rheum. (2011) 63:2091–6. 10.1002/art.30379 21480188

[90] Rueda B Gourh P Broen J Agarwal SK Simeon C Ortego-Centeno N. BANK1 functional variants are associated with susceptibility to diffuse systemic sclerosis in Caucasians. Ann Rheum Dis. (2010) 69:700–5. 10.1136/ard.2009.118174 19815934 PMC2975737

[91] Yoshizaki A. Pathogenic roles of B lymphocytes in systemic sclerosis. Immunol Lett. (2018) 195:76–82. 10.1016/j.imlet.2018.01.002 29307688

[92] Barger SW Hörster D Furukawa K Goodman Y Krieglstein J Mattson MP. Tumor necrosis factors alpha and beta protect neurons against amyloid beta-peptide toxicity: evidence for involvement of a kappa B-binding factor and attenuation of peroxide and Ca2+ accumulation. Proc Natl. Acad Sci U S A. (1995) 92:9328–32. 10.1073/pnas.92.20.9328 7568127 PMC40978

[93] Parks CG Pandey JP Dooley MA Treadwell EL St Clair EW Gilkeson GS. Genetic polymorphisms in tumor necrosis factor (TNF)-alpha and TNF-beta in a population-based study of systemic lupus erythematosus: associations and interaction with the interleukin-1alpha-889 C/T polymorphism. Hum Immunol. (2004) 65:622–31. 10.1016/j.humimm.2004.03.001 15219382

[94] Tolusso B Fabris M Caporali R Cuomo G Isola M Soldano F. 238 and +489 TNF-alpha along with TNF-RII gene polymorphisms associate with the diffuse phenotype in patients with systemic sclerosis. Immunol Lett. (2005) 96:103–8. 10.1016/j.imlet.2004.08.002 15585313

[95] Gourh P Arnett FC Tan FK Assassi S Divecha D Paz G. Association of TNFSF4 (OX40L) polymorphisms with susceptibility to systemic sclerosis. Ann Rheum Dis. (2010) 69:550–5. 10.1136/ard.2009.116434 19778912 PMC2927683

[96] Tan FK Zhou X Mayes MD Gourh P Guo X Marcum C. Signatures of differentially regulated interferon gene expression and vasculotrophism in the peripheral blood cells of systemic sclerosis patients. Rheumatology. (2006) 45:694–702. 10.1093/rheumatology/kei244 16418202

[97] Del Galdo F Maul GG Jiménez SA Artlett CM. Expression of allograft inflammatory factor 1 in tissues from patients with systemic sclerosis and *in vitro* differential expression of its isoforms in response to transforming growth factor beta. Arthritis Rheum. (2006) 54:2616–25. 10.1002/art.22010 16868985

[98] Del Galdo F Artlett CM Jimenez SA. The role of allograft inflammatory factor 1 in systemic sclerosis. Curr Opin Rheumatol. (2006) 18:588–93. 10.1097/01.bor.0000245724.94887.c4 17053503

[99] Otieno FG Lopez AM Jimenez SA Gentiletti J Artlett CM. Allograft inflammatory factor-1 and tumor necrosis factor single nucleotide polymorphisms in systemic sclerosis. Tissue Antigens. (2007) 69:583–91. 10.1111/j.1399-0039.2007.00830.x 17498268

[100] Wu M Skaug B Bi X Mills T Salazar G Zhou X. Interferon regulatory factor 7 (IRF7) represents a link between inflammation and fibrosis in the pathogenesis of systemic sclerosis. Ann Rheum Dis. (2019) 78:1583–91. 10.1136/annrheumdis-2019-215208 31439591 PMC7167109

[101] Rezaei R Mahmoudi M Gharibdoost F Kavosi H Dashti N Imeni V. IRF7 gene expression profile and methylation of its promoter region in patients with systemic sclerosis. Int J Rheum Dis. (2017) 20:1551–61. 10.1111/1756-185X.13175 28952189

[102] Oka A Asano Y Hasegawa M Fujimoto M Ishikawa O Kuwana M. RXRB is an MHC-encoded susceptibility gene associated with anti-topoisomerase I antibody-positive systemic sclerosis. J Investigat Dermatol. (2017) 137:1878–86. 10.1016/j.jid.2017.04.028 28506627

[103] Thomas RM Worswick S Aleshin M. Retinoic acid for treatment of systemic sclerosis and morphea: a literature review. Dermatol Ther. (2017). 10.1111/dth.12455. [Epub ahead of print]. 28032675

[104] Jimenez SA Derk CT. Following the molecular pathways toward an understanding of the pathogenesis of systemic sclerosis. Ann Intern Med. (2004) 140:37–50. 10.7326/0003-4819-140-1-200401060-00010 14706971

[105] Koenig M Joyal F Fritzler MJ Roussin A Abrahamowicz M Boire G. Autoantibodies and microvascular damage are independent predictive factors for the progression of Raynaud's phenomenon to systemic sclerosis: a twenty-year prospective study of 586 patients, with validation of proposed criteria for early systemic sclerosis. Arthritis Rheum. (2008) 58:3902–12. 10.1002/art.24038 19035499

[106] Pavlov-Dolijanovic SR Damjanov NS Vujasinovic Stupar NZ Baltic S Babic DD. The value of pattern capillary changes and antibodies to predict the development of systemic sclerosis in patients with primary Raynaud's phenomenon. Rheumatol Int. (2013) 33:2967–73. 10.1007/s00296-013-2844-7 23934522

[107] Moinzadeh P Nihtyanova SI Howell K Ong VH Denton CP. Impact of hallmark autoantibody reactivity on early diagnosis in scleroderma. Clin Rev Allergy Immunol. (2012) 43:249–55. 10.1007/s12016-012-8331-1 22711501

[108] Valentini G Marcoccia A Cuomo G Vettori S Iudici M Bondanini F. Early systemic sclerosis: marker autoantibodies and videocapillaroscopy patterns are each associated with distinct clinical, functional and cellular activation markers. Arthritis Res Ther. (2013) 15:R63. 10.1186/ar4236 23718566 PMC4060381

[109] Valentini G Marcoccia A Cuomo G Vettori S Iudici M Bondanini F Early systemic sclerosis: analysis of the disease course in patients with marker autoantibody and/or capillaroscopic positivity. Arthritis Care Res. (2014) 66:1520–7. 10.1002/acr.22304 24515450

[110] Srivastava N Hudson M Tatibouet S Wang M Baron M Fritzler MJ. Thinking outside the box–the associations with cutaneous involvement and autoantibody status in systemic sclerosis are not always what we expect. Semin Arthritis Rheum. (2015) 45:184–9. 10.1016/j.semarthrit.2015.04.009 25959492

[111] Nihtyanova SI Sari A Harvey JC Leslie A Derrett-Smith EC Fonseca C. Using autoantibodies and cutaneous subset to develop outcome-based disease classification in systemic sclerosis. Arthritis Rheumatol. (2020) 72:465–76. 10.1002/art.41153 31682743

[112] Sobanski V Giovannelli J Allanore Y Riemekasten G Airò P Vettori S. Phenotypes determined by cluster analysis and their survival in the prospective european scleroderma trials and research cohort of patients with systemic sclerosis. Arthritis Rheumatol. (2019) 71:1553–70. 10.1002/art.40906 30969034 PMC6771590

[113] Igusa T Hummers LK Visvanathan K Richardson C Wigley FM Casciola-Rosen L. Autoantibodies and scleroderma phenotype define subgroups at high-risk and low-risk for cancer. Ann Rheum Dis. (2018) 77:1179–86. 10.1136/annrheumdis-2018-212999 29678941 PMC6272061

[114] Magna M Pisetsky DS. The alarmin properties of DNA and DNA-associated nuclear proteins. Clin Ther. (2016) 38:1029–41. 10.1016/j.clinthera.2016.02.029 27021604

[115] Galluzzi L Vitale I Aaronson SA Abrams JM Adam D Agostinis P. Molecular mechanisms of cell death: recommendations of the nomenclature committee on cell death 2018. Cell Death Differ. (2018) 25:486–541. 10.1038/s41418-017-0012-4 29362479 PMC5864239

[116] Mehta H Goulet PO Nguyen V Pérez G Koenig M Senécal JL. Topoisomerase I peptide-loaded dendritic cells induce autoantibody response as well as skin and lung fibrosis. Autoimmunity. (2016) 49:503–13. 10.1080/08916934.2016.1230848 27808577

[117] Brown M O'Reilly S. The immunopathogenesis of fibrosis in systemic sclerosis. Clin Exp Immunol. (2019) 195:310–21. 30430560 10.1111/cei.13238 PMC6378383

[118] Robitaille G Hénault J Christin MS Senécal JL Raymond Y. The nuclear autoantigen CENP-B displays cytokine-like activities toward vascular smooth muscle cells. Arthritis Rheum. (2007) 56:3814–26. 10.1002/art.22972 17968937

[119] Robitaille G Christin MS Clément I Senécal JL Raymond Y. Nuclear autoantigen CENP-B transactivation of the epidermal growth factor receptor via chemokine receptor 3 in vascular smooth muscle cells. Arthritis Rheum. (2009) 60:2805–16. 10.1002/art.24765 19714638

[120] Hénault J Robitaille G Senécal JL Raymond Y. DNA topoisomerase I binding to fibroblasts induces monocyte adhesion and activation in the presence of anti-topoisomerase I autoantibodies from systemic sclerosis patients. Arthritis Rheum. (2006) 54:963–73. 10.1002/art.21646 16508979

[121] Czömpöly T Simon D Czirják L Németh P. Anti-topoisomerase I autoantibodies in systemic sclerosis. Autoimmun Rev. (2009) 8:692–6. 10.1016/j.autrev.2009.02.018 19393194

[122] Arcand J Robitaille G Koenig M Senécal JL Raymond Y. The autoantigen DNA topoisomerase I interacts with chemokine receptor 7 and exerts cytokine-like effects on dermal fibroblasts. Arthritis Rheum. (2012) 64:826–34. 10.1002/art.33377 21953548

[123] Smeets RL Kersten BE Joosten I Kaffa C Alkema W Koenen H. Diagnostic profiles for precision medicine in systemic sclerosis; stepping forward from single biomarkers towards pathophysiological panels. Autoimmun Rev. (2020) 19:102515. 10.1016/j.autrev.2020.102515 32173517

